# Supplementary figures and images for: Transient ocular hypertension remodels astrocytes through S100B
Source: PLoS One. 2025 Feb 5;20(2):e0313556. doi: 10.1371/journal.pone.0313556 (PMC11798533; doi:10.1371/journal.pone.0313556)

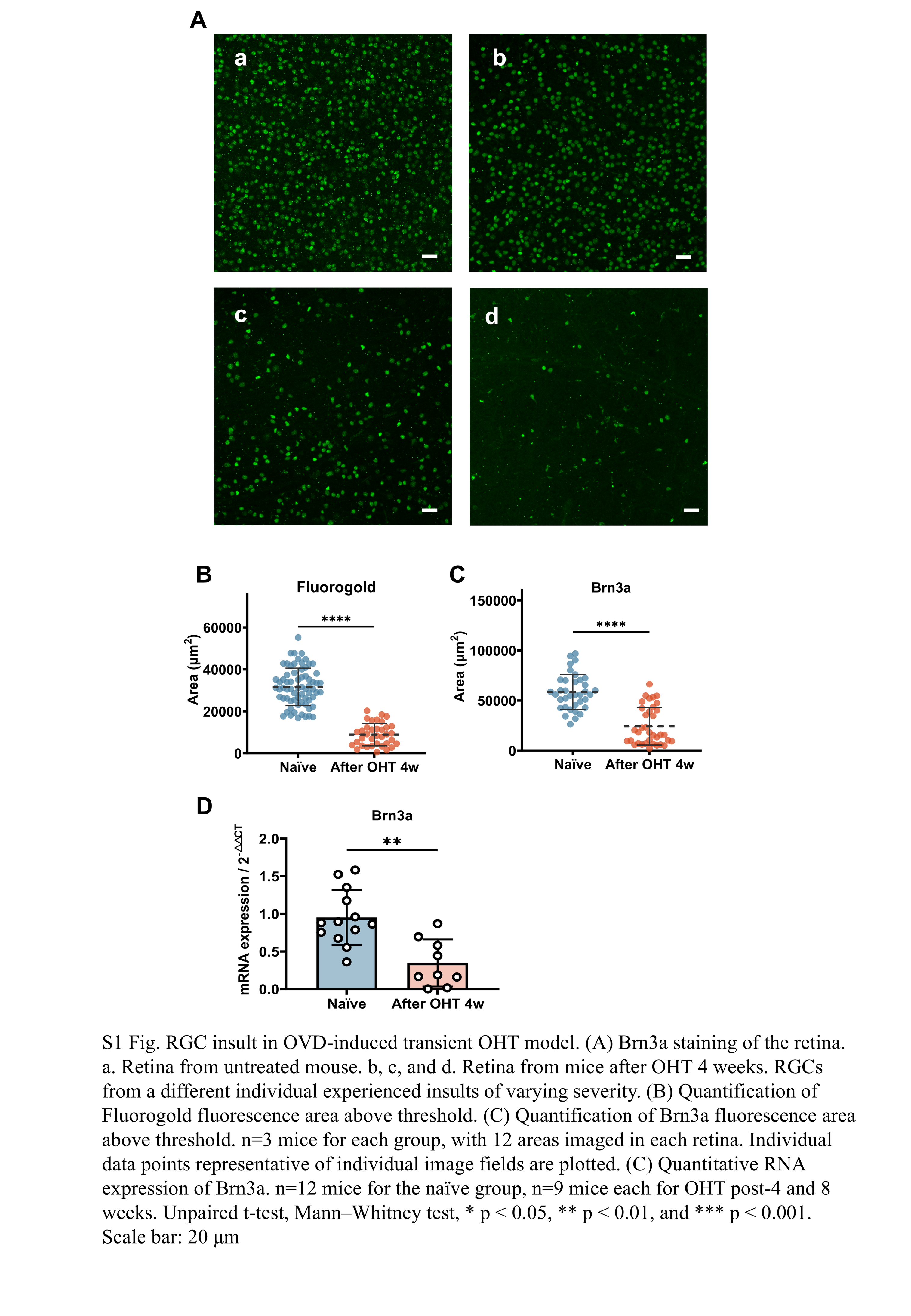

Supplement: S1 Fig — (A) Brn3a staining of the retina. a. Retina from untreated mouse. b, c, and d. Retina from mice after OHT 4 weeks. RGCs from a different individual experienced insults of varying severity. (B) Quantification of Fluorogold fluorescence area above threshold. (C) Quantification of Brn3a fluorescence area above threshold. n = 3 mice for each group, with 12 areas imaged in each retina. Individual data points representative of individual image fields are plotted. (C) Quantitative RNA expression of Brn3a. n = 12 mice for the naïve group, n = 9 mice each for OHT post-4 and 8 weeks. Unpaired t-test, Mann–Whitney test, * p < 0.05, ** p < 0.01, and *** p < 0.001. Scale bar: 20 μm. (TIF) [file pone.0313556.s001.tif]

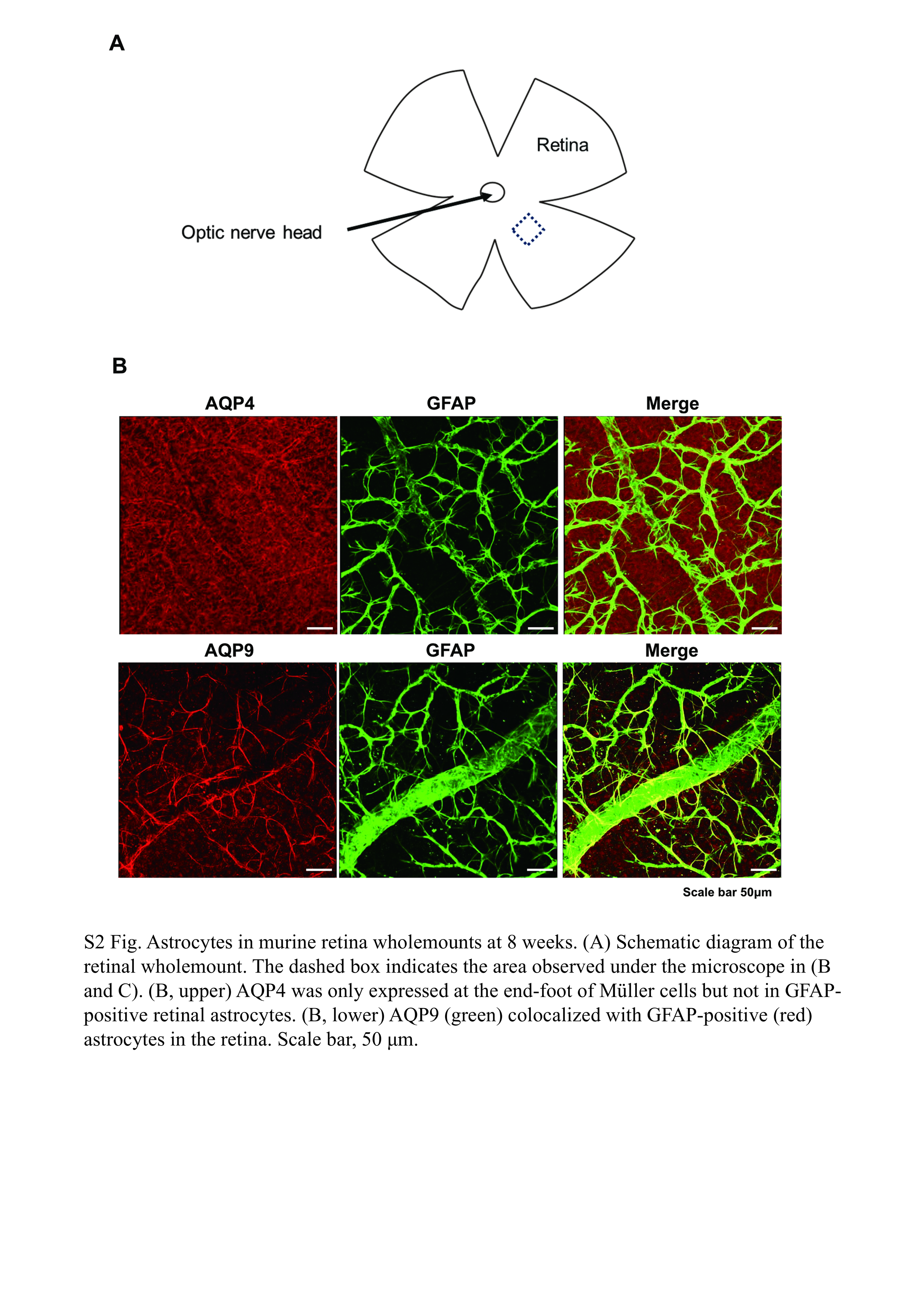

Supplement: S2 Fig — (A) Schematic diagram of the retinal wholemount. The dashed box indicates the area observed under the microscope in (B and C). (B, upper) AQP4 was only expressed at the end-foot of Müller cells but not in GFAP-positive retinal astrocytes. (B, lower) AQP9 (green) colocalized with GFAP-positive (red) astrocytes in the retina. Scale bar, 50 μm. (TIF) [file pone.0313556.s002.tif]

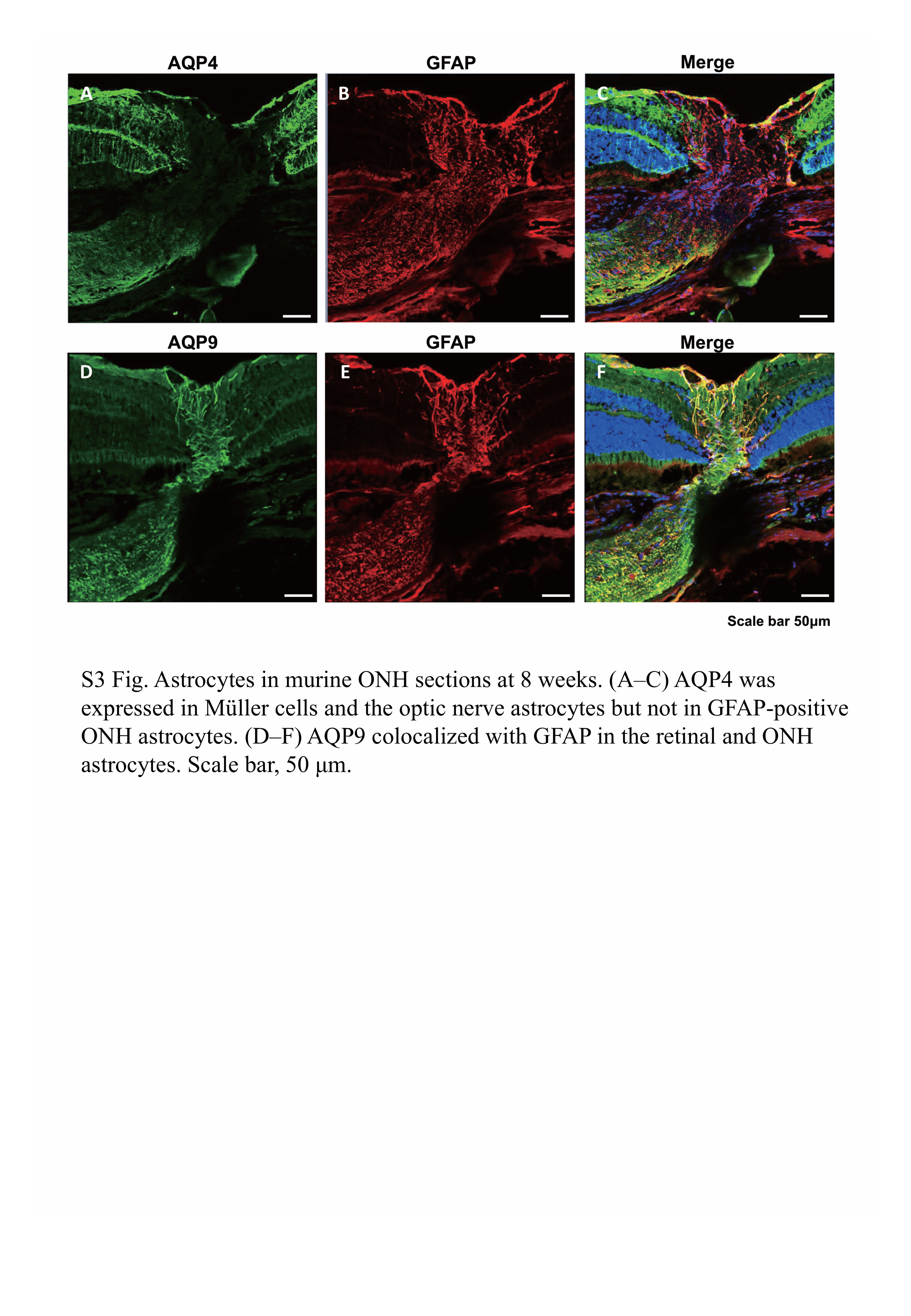

Supplement: S3 Fig — (A–C) AQP4 was expressed in Müller cells and the optic nerve astrocytes but not in GFAP-positive ONH astrocytes. (D–F) AQP9 colocalized with GFAP in the retinal and ONH astrocytes. Scale bar, 50 μm. (TIF) [file pone.0313556.s003.tif]

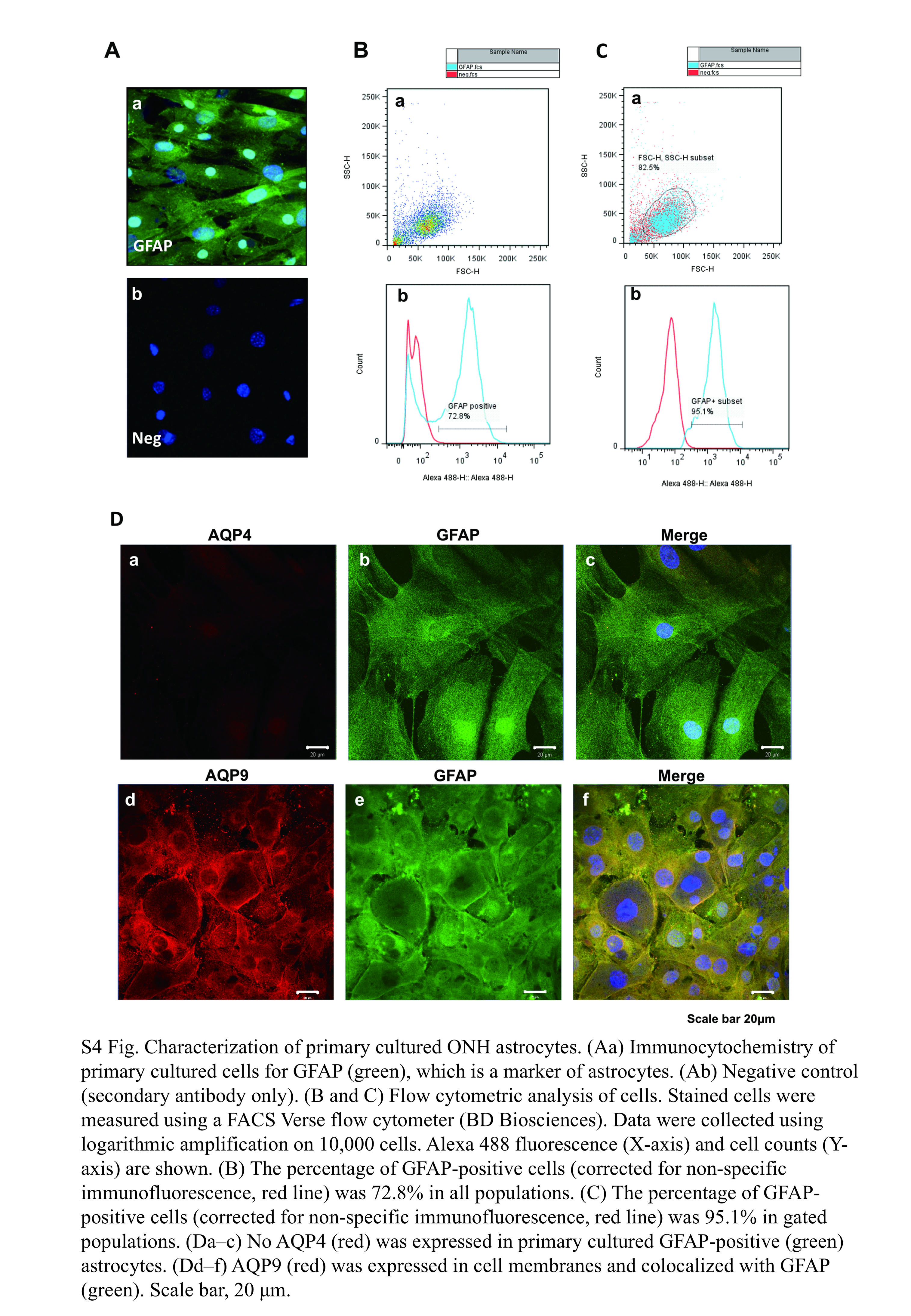

Supplement: S4 Fig — (Aa) Immunocytochemistry of primary cultured cells for GFAP (green), which is a marker of astrocytes. (Ab) Negative control (secondary antibody only). (B and C) Flow cytometric analysis of cells. Stained cells were measured using a FACS Verse flow cytometer (BD Biosciences). Data were collected using logarithmic amplification on 10,000 cells. Alexa 488 fluorescence (X-axis) and cell counts (Y-axis) are shown. (B) The percentage of GFAP-positive cells (corrected for non-specific immunofluorescence, red line) was 72.8% in all populations. (C) The percentage of GFAP-positive cells (corrected for non-specific immunofluorescence, red line) was 95.1% in gated populations. (Da–c) No AQP4 (red) was expressed in primary cultured GFAP-positive (green) astrocytes. (Dd–f) AQP9 (red) was expressed in cell membranes and colocalized with GFAP (green). Scale bar, 20 μm. (TIF) [file pone.0313556.s004.tif]

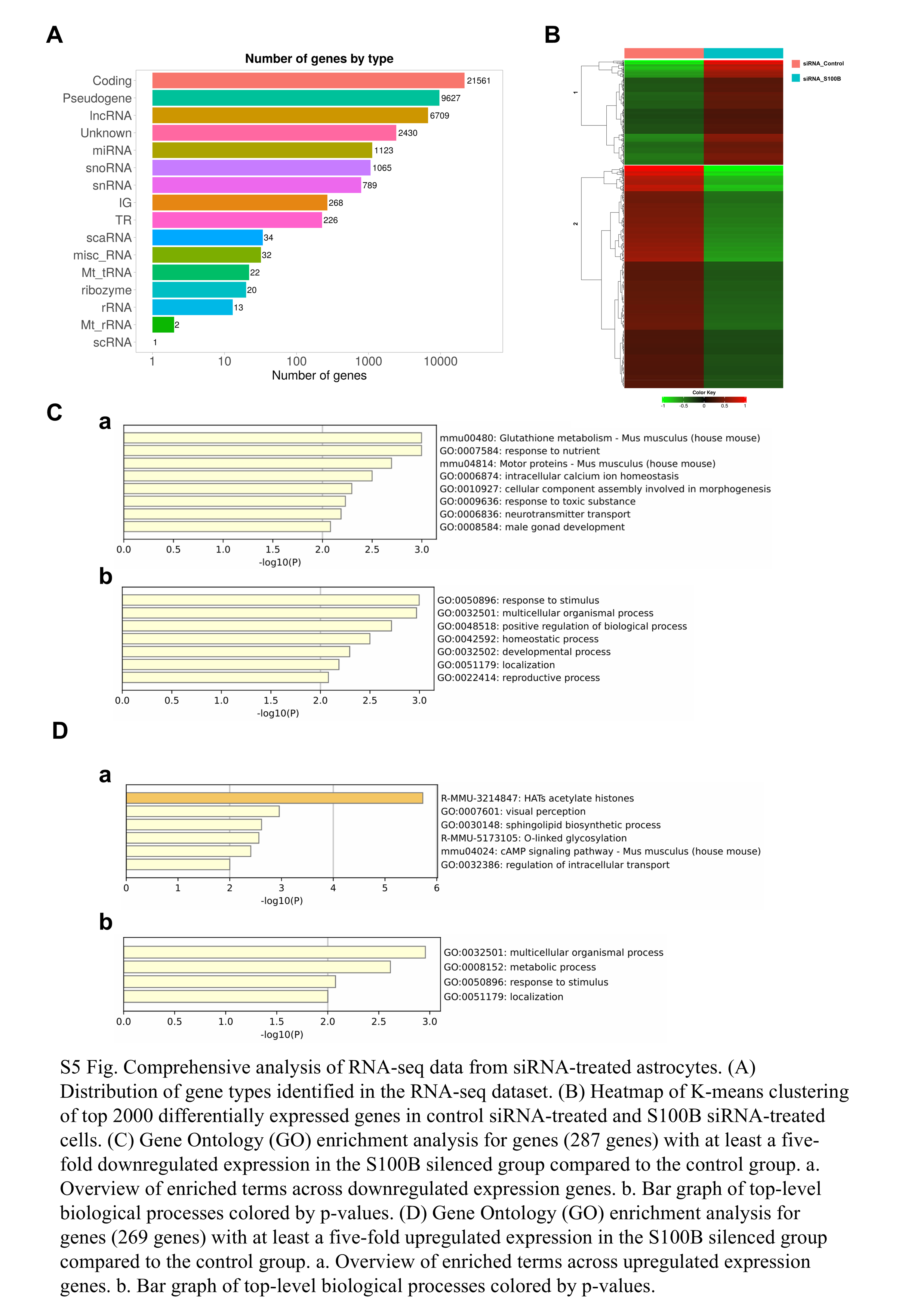

Supplement: S5 Fig — (A) Distribution of gene types identified in the RNA-seq dataset. (B) Heatmap of K-means clustering of top 2,000 differentially expressed genes in control siRNA-treated and S100B siRNA-treated cells. (C) Gene Ontology (GO) enrichment analysis for genes (287 genes) with at least a five-fold downregulated expression in the S100B silenced group compared to the control group. a. Overview of enriched terms across downregulated expression genes. b. Bar graph of top-level biological processes colored by p-values. (D) Gene Ontology (GO) enrichment analysis for genes (269 genes) with at least a five-fold upregulated expression in the S100B silenced group compared to the control group. a. Overview of enriched terms across upregulated expression genes. b. Bar graph of top-level biological processes colored by p-values. (TIF) [file pone.0313556.s005.tif]
